# Evaluating Bicycle Path Roughness: A Comparative Study Using Smartphone and Smart Bicycle Light Sensors

**DOI:** 10.3390/s24227210

**Published:** 2024-11-11

**Authors:** Tufail Ahmed, Ali Pirdavani, Geert Wets, Davy Janssens

**Affiliations:** 1 UHasselt, The Transportation Research Institute (IMOB), Martelarenlaan 42, 3500 Hasselt, Belgium; 2 UHasselt, Faculty of Engineering Technology, Agoralaan, 3590 Diepenbeek, Belgium

**Keywords:** bicycle vibration, comfort assessment, bicycling comfort, smartphone sensors, smart bicycle lights

## Abstract

The quality of bicycle path surfaces significantly influences the comfort of cyclists. This study evaluates the effectiveness of smartphone sensor data and smart bicycle lights data in assessing the roughness of bicycle paths. The research was conducted in Hasselt, Belgium, where various bicycle path pavement types, such as asphalt, cobblestone, concrete, and paving tiles, were analyzed across selected streets. A smartphone application (Physics Toolbox Sensor Suite) and SEE.SENSE smart bicycle lights were used to collect GPS and vertical acceleration data on the bicycle paths. The Dynamic Comfort Index (DCI) and Root Mean Square (RMS) values from the data collected through the Physics Toolbox Sensor Suite were calculated to quantify the vibrational comfort experienced by cyclists. In addition, the data collected from the SEE.SENSE smart bicycle light, DCI, and RMS computed results were categorized for a statistical comparison. The findings of the statistical tests revealed no significant difference in the comfort assessment among DCI, RMS, and SEE.SENSE. The study highlights the potential of integrating smartphone sensors and smart bicycle lights for efficient, large-scale assessments of bicycle infrastructure, contributing to more informed urban planning and improved cycling conditions. It also provides a low-cost solution for the city authorities to continuously assess and monitor the quality of their cycling paths.

## 1. Introduction

A growing body of literature acknowledges the societal advantages of improved bicycle infrastructure [1,2,3]. Bicycle facility planning, construction, and improvement are costly and time-consuming processes. Cyclists should use these facilities; otherwise, it wastes the time and resources of the government. Studies have shown that various factors affect cyclists’ route choices, including environmental conditions, infrastructure, safety, and personal preferences [4,5,6,7,8]. Bicycle routes and lanes should be smooth and undemanding, requiring minimum effort [9,10]. In addition, cyclists consider the pavement condition of a bicycle path extremely important as the quality directly affects their comfort [11,12].

Bicycle vibrations frequently result from poor cycling track conditions, posing a significant challenge for cyclists. Increased vibration levels lead to decreased cycling comfort, with higher speeds and rougher road surfaces exacerbating the issue [13,14]. An uneven pavement is one of the main reasons for vibrations on bicycle paths [14,15]. Research also shows that cyclists perceive vibration as an influencing factor affecting their mode and route choices [16]. Cyclist exposure to vibration can affect their body, particularly in the joints of the lower limbs, potentially leading to health issues [17].

Several studies have investigated the effect of different types of pavements, i.e., asphalt, concrete, paving slabs, and cobblestones, based on bicycle vibration or vertical acceleration with asphalt showing higher comfort [9,16]. Various methods have been utilized to assess bicycle comfort for cycling infrastructure. Traditionally, city authorities use direct visual observation to assess the condition of bicycle pavement, but this technique is very time-consuming and labor-intensive [11]. In addition, this technique requires significant attention to detail from the surveyor, which can further complicate the surveying process [18]. More efficient and objective methods are needed to collect and process data accurately.

Some of the other techniques include the DCI, International Roughness Index (IRI), and RMS. The DCI is a quantitative measure used to assess the comfort of cycling infrastructure by evaluating the vibrations experienced by cyclists. This index is derived from data collected using bicycles equipped with GPS devices and accelerometers [15]. The DCI has been shown to have a strong negative correlation with subjective comfort assessments, indicating its reliability as an objective measure of cycling comfort. The IRI, adapted from a quarter-car model, is used to assess roughness for bicycle paths [19]. Similarly, the RMS has also been used to quantify cycling comfort, especially by characterizing vertical accelerations [20]. Other research determined that a simple way to assess rider comfort is by calculating the RMS value of acceleration, which serves as a comfort indicator [21]. In our previous study, we used SEE.SENSE portable bicycle lights to evaluate cycling comfort based on the vibration on various bicycle infrastructure surfaces in Hasselt, Belgium [16].

Studies have used instrumented probe bikes (IPBs) (i.e., equipped with sensors) to collect data for measuring the comfort of bicycle route pavements. An IPB fitted with sensors, such as a GPS receiver, accelerometer, and video camera, was used to collect data in Singapore [18]. Similarly, another study used an instrumented bicycle equipped with a GPS device and accelerometer to collect data for the DCI [22]. One study used a bicycle with a smartphone attached to the frame to measure vertical acceleration and a GPS for georeferencing [20]. Another recent study used an instrumented city bicycle to collect precise data on the bicycle dynamics, trajectory, and speed [23]. These studies demonstrate the effectiveness of using IPBs to evaluate pavement conditions and surface vibrations, offering accurate measurements. However, IPBs are usually considered expensive due to the high cost of advanced sensors [24].

Mobile technology is crucial for understanding transport behavior, utilizing data from smartphone apps, GPS, and cellular signals [25,26,27]. Also, smartphone apps have emerged as viable options for collecting data from citizens, including transport system users, using GPS records in these devices [28,29]. An illustrative example is CycleTracks, a smartphone application designed by the San Francisco County Transportation Authority, California, for collecting GPS and survey data about cyclists [30]. Similarly, the Strava smartphone app has been used in research to collect information like bicycle volumes for links, nodes, and origin-destination [31]. Other research utilizes GPS tracking data from the Endomondo fitness tracker app for trips in Miami-Dade (Florida) and North Holland [32]. Likewise, another study utilized the Mon RésoVélo smartphone application to collect GPS cyclist trip data. Studies using smartphone-based methods have demonstrated the feasibility of using smartphone devices for large-scale data collection, making understanding cyclist behavior and infrastructure use easier. These methods offer scalable, accessible solutions that can reach a broader audience than traditional instrumented bikes.

Smartphone sensors have also been used to assess cyclists’ comfort by collecting data, such as vibrations on bicycle paths [12,19,33,34]. One study developed an innovative Dynamic Cycling Comfort system, consisting of an accelerometer, GPS logger, and smartphone (VBOX app), to collect and analyze data on vibration, speed, and mileage during cycling trips [12]. Another study employed the Phyphox app to gather similar data on vibrations and cycling conditions, further validating the use of smartphone-based sensors in comfort assessments [35]. Furthermore, smartphones mounted on bicycles have been utilized to measure surface roughness on roads inaccessible to traditional motor vehicles, such as pedestrian and bicycle lanes, by developing a dedicated app, RoadSR [19]. In addition to employing smartphone applications like the VBOX and Phyphox for vibration data collection, another dedicated Android app called RideVibes was developed [33]. Moreover, one study used the VTI app developed by the Swedish National Road and Transport Research Institute to find the relationship between the cyclists’ subjective evaluations of riding comfort and the accelerometer measurement from the app [36]. These studies have established that smartphone app-based methods are reliable and effective for measuring vibrations, offering an inexpensive alternative to IPBs.

Recent studies have shown the potential of using smart bicycle lights to collect vibration data [16,34]. One study used portable smart bicycle lights (SEE.SENSE ACE) to evaluate the vibration levels on cycling infrastructure, which are used to assess cycling comfort [16]. The smart bicycle lights capture a three-axis accelerometer capturing up to 800 readings per second, processed with AI [37]. It also collects ride data, such as vibration, location, speed, swerve, etc. A subjective assessment showed that cyclists’ comfort is inversely related to the vibration experienced on different surfaces. Similarly, another study used crowdsourced datasets from Findrs smart bicycle lights to calculate the surface roughness using the DCI method [34]. A secondary dataset from Hövding (bicycle airbag helmet) was also used, containing data like User ID information, coordinates, timestamps, etc. Both the devices were connected to the smartphone apps. While not as advanced as IPBs, these smart bicycle lights provide relevant data at a lower cost, making them accessible for large-scale data collection.

Over the years, various methods have been used to measure cycling comfort, including IPBs, smartphone applications, and smart bicycle lights. Due to their advanced sensors, instrumented bikes offer precise data but are costly. Alternatively, smartphone apps and smart bicycle lights deliver extendable, affordable solutions to data acquisition. In addition, several approaches, such as DCI and RMS, and smart bicycle light sensors like SEE.SENSE have been used individually to assess cycling comfort. Smart bicycle lights serve a dual purpose: they function as regular lights for safe riding while aiding data collection. Paired with a smartphone, the app captures vibration, swerve, and braking data, which are useful for city authorities and also provide riders with insights and benefits from their cycling activities. SEE.SENSE is a low-cost alternative with the added advantage of integrating into city bicycles to capture surface roughness data. These data, which can be collected citywide through crowdsourced usage, hold significant potential for providing city authorities with ongoing, scalable, and detailed insights into road surface conditions. Such data could help monitor cycling surface deterioration and prioritize maintenance over time. However, to ensure the reliability, SEE.SENSE must be validated against established methods like DCI and RMS. Little attention has been given to comparing the accuracy and consistency of cycling comfort methods under various cycling surface conditions. In addition, we could not find a study that compared the results of different techniques and verified the validity of SEE.SENSE, against other established methods. Therefore, this study aims to compare results from different cycling comfort assessment methods on various pavement types and assess the validity of the results obtained from using SEE.SENSE devices.

## 2. Materials and Methods

### 2.1. Assessments of Street Surface in the Study Area

Table 1 shows the selected streets length and pavement type in the study area. This study considered various street pavement types to assess cyclist comfort. The sample includes two asphalt streets, allowing for the evaluation of this common and typically smooth surface. Three concrete paved streets are considered, and three mixed paved streets (asphalt and concrete streets, cobblestone and paving tiles, and asphalt and paving tiles) are used to provide the opportunity to study how the comfort on these streets could vary as both pavement types produce different vibrations. Three streets with paving tiles are examined, which may present different textures and joint patterns. Three cobblestone streets are part of the selected streets, challenging surfaces that create much vibration and can affect the cycling experience and comfort. The pavement condition of the selected streets can be seen in Appendix A.

### 2.2. Data Collection

Mobile applications can take advantage of the many sensors available on the smartphone, such as accelerometers, gyro, and GPS [36]. The surface irregularities generate vibration measured by the smartphone accelerometers that can be used to assess comfort [14,36]. Multiple smartphone applications were initially considered for collecting vibration data, as shown in Table 2. While most applications are free, some charge for additional pro features. For example, exporting combined data to CSV in the Sensor Logger app requires a pro plan (3.99 Euros per month), and the SensorLog app must be purchased for 5.99 euros. The Phyphox App is also free; however, it does not support the combined export of GPS and acceleration data, making it less suitable for locational vibration data collection on road or bicycle paths. Additionally, the Sensors Toolbox-Multitool app and EXA Sensors Toolbox do not export combined data. The EXA Sensors Toolbox must be purchased (1.99 Euro) to export data. Also, another limitation of the Sensors Toolbox-Multitool app is that it only exports the data in plain text, which is not useful for later analysis. Similarly, Physics Toolbox Accelerometer is a dedicated app for three-dimensional accelerometer data but does not record the location data. In Table 2, a green check mark indicates the presence of a feature, while a red cross mark denotes its absence.

After evaluating the options, the Physics Toolbox Sensor Suite app downloaded from the IOS app store (version date 14/08/2024) developed by Vieyra software (https://www.vieyrasoftware.net/, accessed on 3 October 2024) was installed on a smartphone mounted on the bicycle using a phone holder. This app was chosen because it simultaneously collects GPS and vertical acceleration data, which can be used for analysis and visualization in a GIS environment. In addition, the app is free to use and does not require a subscription to export the data. Also, the app offers a combined data exporting (the location and accelerometer data) option in CSV.

The data collection process involved a cyclist riding along the selected streets with a smartphone mounted on a bicycle. The smartphone was mounted on the handlebar of a regular cycle. The handle is considered the most sensitive contact point for detecting vibrations. The smartphone app continuously recorded vibration data (accelerometer readings) and GPS locations during the ride.

Smart bicycle lights developed by SEE.SENSE (a company based in Newtownards, UK) can gather cycling vibration data. For this study, we have used the SEE.SENSE ACE portable bicycle light model. These intelligent lights can record vibration values on a scale from 0 to 100, where lower values indicate smoother bicycle path surfaces, while higher values signify rougher surfaces that generate more vibrations. SEE.SENSE utilizes crowdsourced sensor data processed with edge computing and artificial intelligence to assess road surface quality [37].

The smartphone application with these bicycle lights automatically associates the data with GPS coordinates. This combination of GPS-linked data enables the identification of problematic surface conditions along cycling routes. The data collection setup involves attaching two lights to a standard bicycle, one at the front and one at the rear. These smart lights can be connected to a smartphone via the SEE.SENSE app. The smartphone app automatically pairs these readings with GPS locations, enabling them to visualize the vibration data of the collected streets.

The setup process is simple and involves three main steps:

- Mount the SEE.SENSE lights on the front and rear of the bicycle;
- Connect both lights to the app via Bluetooth;
- Activate the lights using the app or physical button. Data collection begins automatically when the lights are turned on and stops when switched off.

In our previous study, we assessed vibration sensitivity across two cycling paths (different pavements) along Hasselt’s Inner Ring (R70), comparing a newly built with an older bicycle path [16]. The SEE.SENSE sensitivity tests were conducted with six riders, and the results showed no notable differences in vibration measurements along both selected bicycle paths. The smart bicycle lights reliably detected vibration across the surfaces tested.

Studies have shown that cyclists’ travel speeds could significantly impact the surface roughness index results [15,34]. Hence, the riders were asked to maintain a speed between 13 and 15 km/h during the data collection to control its potential impact on vibration values. The previous research suggests that this approach helps avoid the influence of speed on vertical acceleration values [33,36]. The data on the Physics Toolbox Sensor Suite app were collected at a sampling rate of 100 Hz. After the completion of the data collection, the vibration data and GPS data from the SEE.SENSE and Physics Toolbox Sensor Suite app were exported. The data, including the GPS data and vertical acceleration values, were exported to a suitable format (.CSV) for further processing and analysis. The data analysis was carried out using Python (version 3.10).

### 2.3. Data Processing

After collecting the data using the app, the dataset was processed by calculating distances based on sequential GPS coordinates (latitude and longitude). The Haversine formula can estimate the distance between two sampling points (See Equation (1)).

(1) $$d=2\;*\;r\;*\;arcsin\left(\sqrt{{sin}^{2}\left(\frac{\varphi_{2}-\varphi_{1}}{2}\right)+cos(\varphi_{1})\;*\;cos(\varphi_{2})\;*\;{sin}^{2}\left(\frac{\lambda_{2}-\lambda_{1}}{2}\right)\;}\right)$$

where *d* is the distance between the two points, and *r* is the radius of the Earth (mean radius is approximately 6371 km). $\varphi_{1}\;$ and $\varphi_{2}$ are the latitudes of the two points, $\lambda_{1}$ and $\lambda_{2}$ are the longitudes of the two points.

Using the Haversine formula, distances between points were computed for each street, and the data were then divided into 10-m segments. For each 10-m segment, the DCI, RMS, and SEE.SENSE values were calculated, and their mean values were categorized from 1 to 5. These categorized metrics were subsequently used for statistical analysis.

### 2.4. Dynamic Comfort Index

In our study, we used the DCI proposed by Bíl et al. [15] to objectively quantify the vibrational comfort of cycling tracks. The DCI is computed from acceleration data captured by the smartphone app mounted on a bicycle. This index serves as an inverse measure of the vibrational energy experienced by cyclists, providing a numerical value that reflects the comfort level of the surface pavement. The DCI can be calculated using Equation (2).

(2) $$\mathrm{DCI}={\left(\sqrt{\frac{1}{n}\;\sum_{i=1}^{n}{a_{i}}^{2}}\right)}^{-1}$$

where (*n*) represents the number of acceleration measurements exceeding one *g* (vertical gravitational acceleration) recorded for each geographic location, and *a* is the vertical acceleration value showing the bicycle vibration on the ridden paths or streets. The DCI value falls between zero and one. The DCI quantifies the comfort level of a bicycle ride by measuring vibrations using acceleration data. The higher DCI values on a bicycle surface indicate that bicycle paths or lanes are more comfortable and have less vibration. In contrast, lower DCI values correspond to roads with higher vibration levels. The DCI values, ranging from 0 to 1, can be converted to a comfort scale of 1 to 5 for statistical tests, where 1 represents “extremely comfortable” and 5 indicates “extremely uncomfortable.” Scaling the data from 1 to 5 provides a more understandable interpretation, with 5 representing the lowest level of comfort and 1 the highest, making the results easier for statistical analysis and comparisons.

### 2.5. Root Mean Square

The analysis of vibration levels experienced by the cyclist’s body due to road conditions can be based on the guidelines provided by the International Organization for Standardization (ISO) 2631-1 [38]. The RMS has been previously utilized in bicycle-path-pavement assessment studies [12,20,38]. To calculate the RMS, we used vertical acceleration, the z-axis data captured by the smartphone app, as this is the primary way cyclists perceive surface irregularities on the pavement. Equation (3) is used to calculate the RMS.

(3) $$\mathrm{RMS}=\sqrt{\frac{1}{n}\sum_{i=1}^{n}{x_{i}}^{2}}$$

where *n* represents the number of measurements, and $x_{i}$ is the vertical acceleration values recorded along the path.

The comfort for cyclists can be determined after calculating the RMS for each street using Table 3. The first column of Table 3 indicates that ISO 2631-1 allows for class overlap. We adopted a similar approach used by Nuñez et al. [20] to classify the likely users’ reactions (refer to Table 3). This is performed to avoid considering overlapping values and to ensure that no values are simultaneously classified into two categories. Additionally, we classified them into five categories for a comparison and statistical analysis.

### 2.6. SEE.SENSE Comfort Rating

Cyclists’ comfort perception was assessed through questionnaires, revealing a strong positive correlation (Pearson correlation coefficient 0.91) between measured vibration levels using SEE.SENSE and subjective comfort ratings [16]. The study categorized cycling comfort based on vibration levels: <4 as “extremely comfortable”, 4–10 as “somewhat comfortable”, 11–15 as “neither comfortable nor uncomfortable”, >15 as uncomfortable, and >26 as “extremely uncomfortable”. We will use these categories in the analysis, as established in our previous study [16].

### 2.7. Statistical Analysis

We conducted non-parametric statistical tests to evaluate the differences in bicycle comfort assessment across various streets using three methods (SEE.SENSE, DCI, and RMS). Non-parametric tests do not assume that a specific data distribution is well-suited for ordinal data like our comfort ratings [39]. Since the comfort categorization is ordinal (1–5) in our case, using the mean or standard deviation is unsuitable, meaning that statistical methods, such as the *t*-test, F-test, and ANOVA—typical parametric approaches—are not appropriate for analyzing ordinal data [40]. Specifically, we used the Wilcoxon Signed-Rank Test to compare the paired comfort assessments between each combination of the three methods, i.e., SEE.SENSE and DCI, SEE.SENSE and RMS, and DCI and RMS. This test was selected due to the nature of our data: we categorized comfort on an ordinal scale from 1 to 5 based on established guidelines from the literature. This test identifies whether the median differences in comfort ratings between each pair of methods are statistically significant, which helps us understand if the methods yield similar results on a paired basis.

In addition, we also performed the Friedman Test, which assesses differences across more than two groups. The Friedman Test is a non-parametric alternative to ANOVA and is useful when evaluating differences across multiple methods (more than 2). In our study, this test examines whether comfort assessments differ significantly when considering all three methods (SEE.SENSE, DCI, and RMS) together. This test allows us to evaluate consistency across methods in one analysis. Both tests were conducted at the 5% significance level to assess whether the methods produced significantly different comfort categorizations. 

To evaluate the consistency of the comfort assessments using different methods across different streets, we formulated the following hypotheses:

**H0:**  *There is no statistically significant difference in the comfort assessment of streets among the SEE.SENSE Comfort, DCI, and RMS methods.*

**H1:**  *There is a statistically significant difference in the comfort assessment of streets among the SEE.SENSE Comfort, DCI, and RMS methods.*

## 3. Results

**Figure 1:**
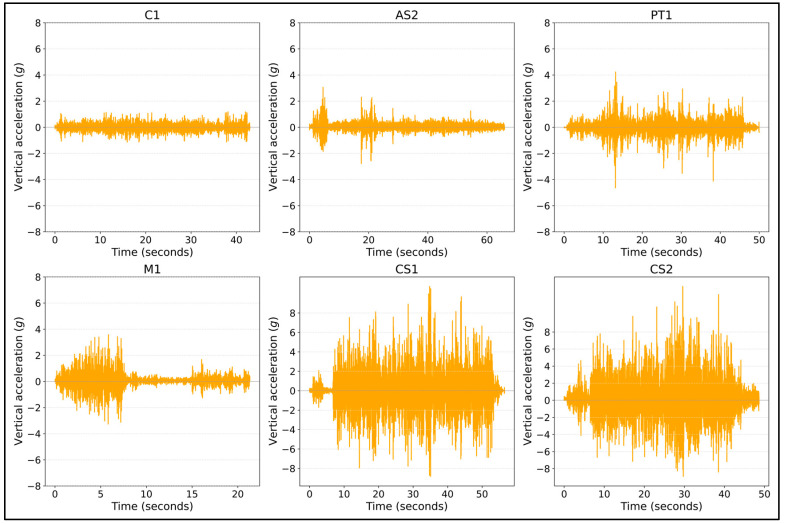
Unprocessed data from the Physics Toolbox Sensor Suite application.

Figure 1 presents six plots, each representing the unprocessed vertical acceleration captured using the Physics Toolbox Sensor Suite app across different bicycle streets: C1, AS2, PT1, M1, CS1, and CS2. The data points in these plots reflect the vertical acceleration values obtained along each street. Figure 1 illustrates the variability in surface conditions, with streets like M1, CS1, and CS2 showing higher fluctuations in vertical acceleration, indicating rougher surfaces or more significant disturbances. Meanwhile, C1 and AS2 exhibit more stable profiles, suggesting smoother surfaces.

**Figure 2:**
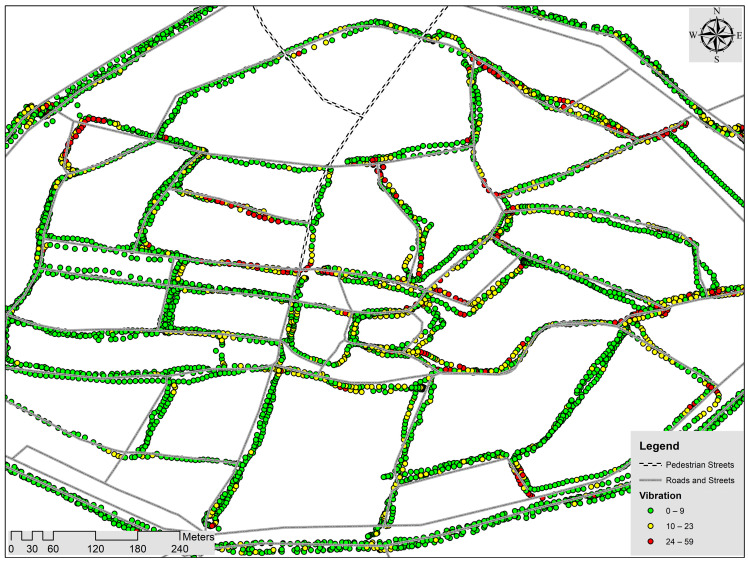
Vibration data from the SEE.SENSE smart bicycle lights.

Similarly, Figure 2 displays the vibration data captured using SEE.SENSE smart bicycle lights. To understand the values better, we have used ArcMap (version 10.1) license obtained through Uhasselt Belgium from the Environmental System Research Institute headquartered in Redlands, California, to visualize the data. Three colors have been used for visualization: the green color shows the low vibration values, the yellow color shows medium, and the red color depicts the high vibration values on the ridden bicycle path.

**Figure 3:**
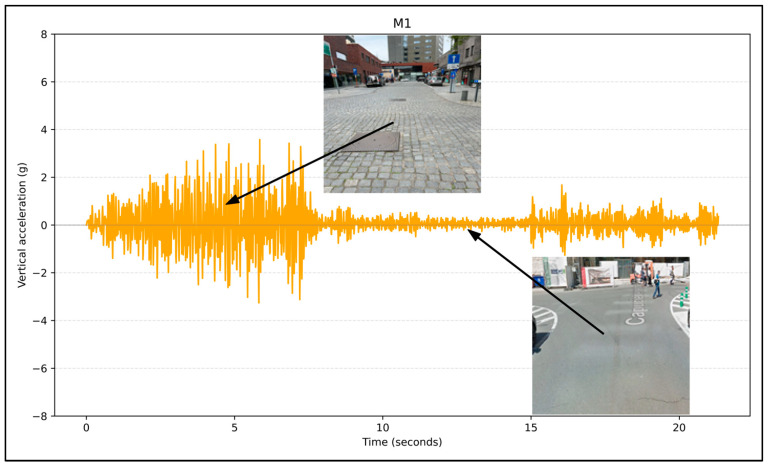
Acceleration on bicycle streets with different surface pavements (asphalt-paved and cobblestone-paved).

**Figure 4:**
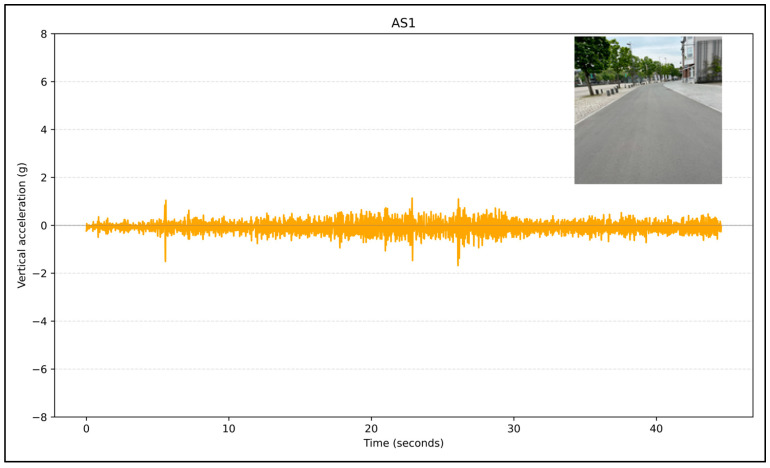
Acceleration on asphalt-paved bicycle streets.

**Figure 5:**
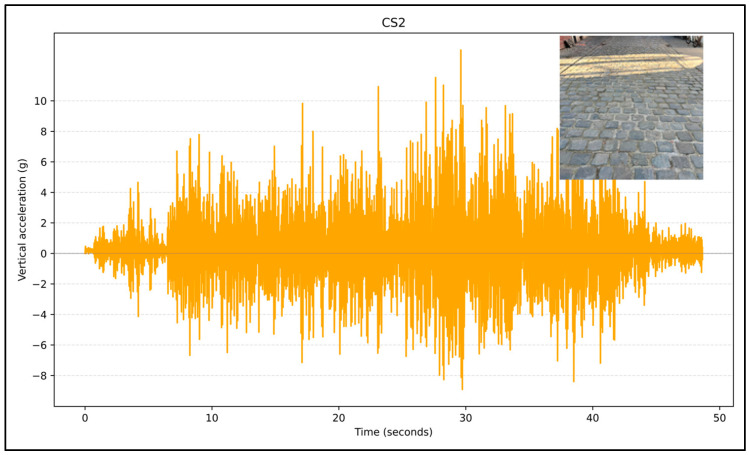
Acceleration on cobblestone-paved bicycle streets.

The vibration recorded via the smartphone application was also checked to see the vertical acceleration data concerning bicycle road surface conditions and measured accelerations. To highlight how surface roughness influences vertical acceleration, we examined streets with varying pavement types, such as asphalt-paved, cobblestone-paved, and mixed-paved bicycle streets. Figure 3, Figure 4 and Figure 5 show the raw data captured on different pavement surfaces. As expected, smoother surfaces result in lower acceleration values, and rougher surfaces result in higher values. Figure 3 represents vertical acceleration measurements across sample points on street M1, indicating road surface conditions—the figure highlights locations with uneven cobblestones and smoother asphalt, correlating with higher and lower acceleration spikes, respectively.

Figure 4 displays vertical acceleration data for AS1. The figure indicates a smooth road surface, which correlates with the relatively stable and low vertical acceleration values, suggesting minimal road unevenness.

Figure 5 shows vertical acceleration data for CS2, which features a cobblestone-paved street (as seen in the figure). The vertical vibration values on this street are very high, indicating a rough pavement. This roughness leads to significant vertical acceleration variations. Figure 3, Figure 4 and Figure 5 indicate that the vertical acceleration values correlate strongly with road surface conditions. Smoother surfaces like asphalt result in lower and more stable vertical accelerations, while uneven surfaces, such as cobblestones, causing high values.

### 3.1. DCI Values

**Figure 6:**
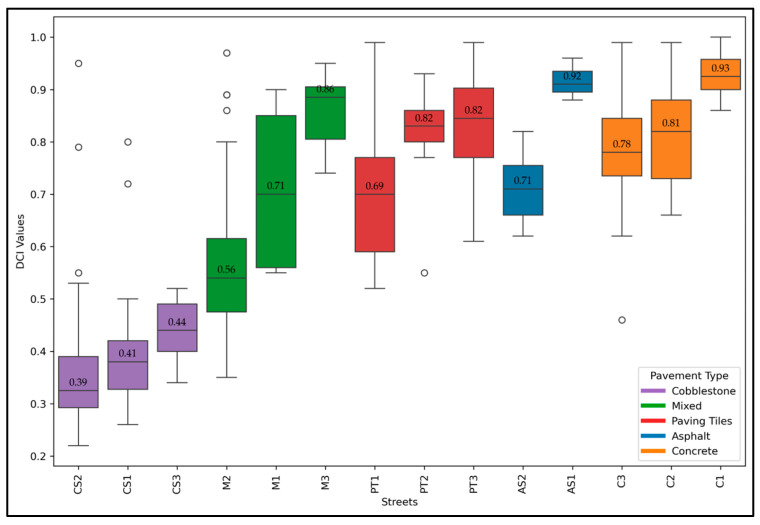
DCI of study area bicycle streets.

Figure 6 shows the DCI values computed for the streets in the study area. The vertical acceleration collected using the Physics Toolbox Sensor Suite app was processed using Equation (1) to compute the DCI. The DCI values across various streets in the study area highlight significant differences in cyclist comfort. AS1 (asphalt-paved street) and C1 (concrete-paved street) showed the highest mean DCIs of 0.92 and 0.93 with minimal variation, indicating that they provide the smoothest cycling experience among the studied locations.

In contrast, CS1 and CS2, both cobblestone-paved streets, recorded the lowest mean DCI values of 0.41 and 0.39, respectively, reflecting a much rougher and less comfortable ride for cyclists. M1, which features both asphalt and cobblestone pavements, showed a mean DCI of 0.71, suggesting good comfort with some variability due to the mixed pavement types. PT2 and PT3, primarily paving tile-paved, also scored high on comfort with mean DCI values of 0.82.

### 3.2. RMS Values

**Figure 7:**
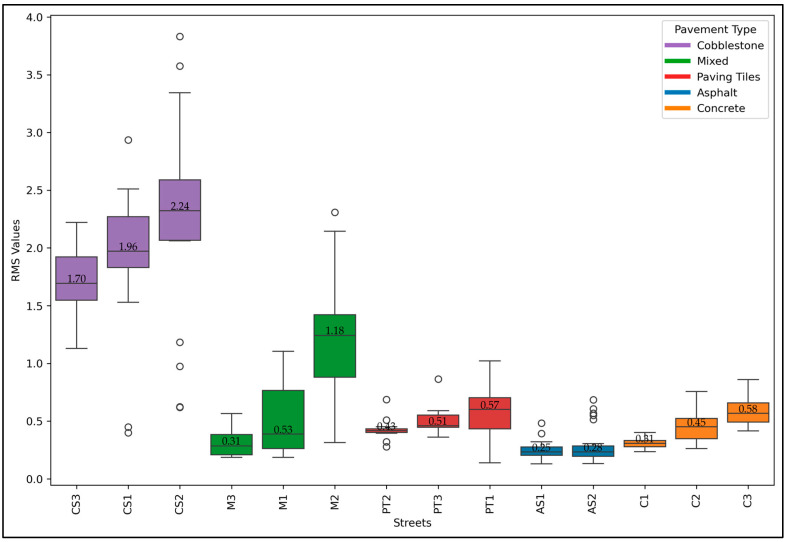
RMS of study area bicycle streets.

The RMS values for the case study area are reported in Figure 7. The RMS was computed using the vertical acceleration collected using the Physics Toolbox Sensor Suite app and was processed using Equation (2). The RMS values can be used to measure the intensity of vibrations experienced by the cyclists provided. CS2 had the highest RMS value at 2.24. Other streets with very high RMS values are CS1, with an RMS value of 1.96, and CS3, with an RMS value of 1.70. The AS1 reports the lowest RMS value at 0.25. M3 and M1 also show low RMS values of 0.31 and 0.53, respectively. Streets like AS2, C1, PT2, and C2 display lower RMS values (0.28, 0.31, 0.43, and 0.45, respectively).

### 3.3. SEE.SENSE Values

**Figure 8:**
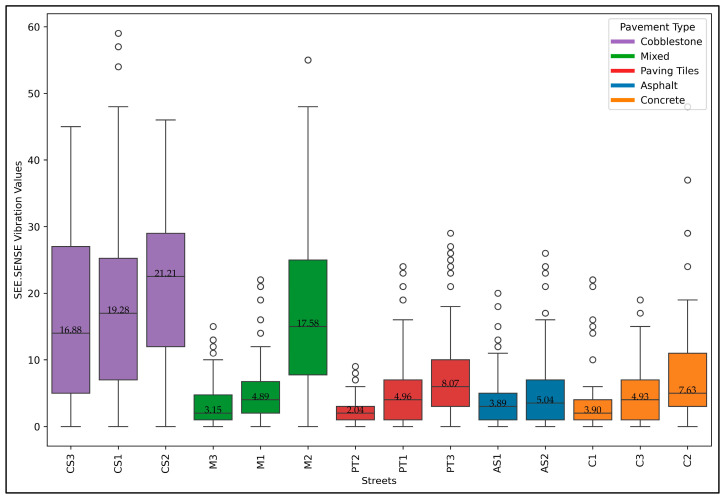
SEE.SENSE vibration values of study area bicycle streets.

Figure 8 shows significant variability in the vibration values obtained using SEE.SENSE. Similar to the DCI and RMS, the streets CS2 and CS1 exhibit higher median vibration values and greater dispersion, suggesting poor pavement conditions and higher discomfort for cyclists. On the other hand, streets like AS1, C1, and PT2 have lower vibration values, indicating smoother surfaces and a more comfortable riding experience.

### 3.4. Comparison of Results

**Figure 9:**
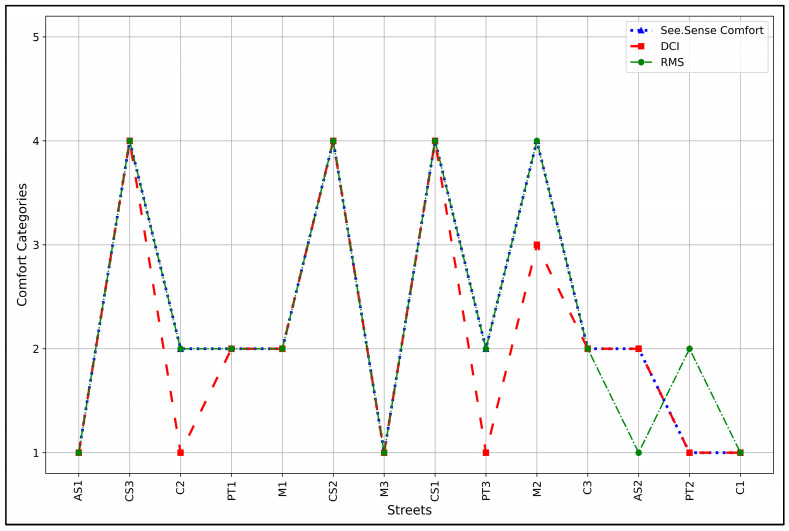
RMS, DCI, and SEE.SENSE of study area bicycle streets.

Figure 9 shows bicycle comfort categories across various streets using three different assessment methods: SEE.SENSE Comfort, DCI, and RMS. The x-axis lists different streets, while the y-axis represents comfort categories on a scale from 1 to 5, where 1 indicates “Extremely comfortable” while 5 represents “extremely uncomfortable”. Figure 9 shows comfort levels across streets, with most streets showing agreement between methods without significant discrepancies. We employed the Wilcoxon Signed-Rank Test to see if there was a significant difference between the comfort assessment obtained from the methods used. The results of the Wilcoxon tests comparing SEE.SENSE Comfort and DCI yielded a test statistic of 0 and a *p*-value of 0.083, which is close to but not quite reaching the conventional significance threshold of 0.05. This suggests no significant difference between these methods at the 5% level.

Similarly, the comparison between SEE.SENSE Comfort and RMS produced a statistic of 1.5 with a *p*-value of 1.0, indicating no statistically significant difference between these two methods. Lastly, the test between the DCI and RMS resulted in a statistic of 3.0 and a *p*-value of 0.180, again indicating no statistically significant difference. These results imply that while the three methods show some variations, they do not differ significantly in their overall assessment of comfort across the streets. We also performed the Friedman test to see if there was a statistical difference among the results obtained, taking the results from the three methods together. The Friedman test yielded a *χ²* of 3.60 and a *p*-value of 0.165. The *p*-value was above the common significance level of 0.05, indicating that, at the 5% significance level, there is not enough evidence to reject the null hypothesis. There are no significant differences in the comfort categorization between the methods. This suggests that, for the studied cases, the comfort assessment performed by SEE.SENSE Comfort, DCI, and RMS are analogous.

## 4. Discussion

Cyclists regard the condition of a bicycle path’s pavement as highly important, as its quality directly impacts their comfort [15,41]. Various methods have been developed to assess cyclist comfort based on the vibration induced by pavement irregularities. This study aimed to evaluate and compare the effectiveness of three different methods—SEE.SENSE, DCI, and RMS, which are used to measure comfort based on vibrations experienced by cyclists across various pavement types. Selecting various bicycle path pavement surfaces enables an assessment of whether the three methods can accurately represent various road surfaces and how they perform under different data collection methods (SEE.SENSE bicycle lights and smartphone app). The results provide important findings in evaluating cycling comfort across different streets using different data collection methods. Streets paved with asphalt (very good condition), such as Kolonel Dusartplein (AS1), consistently recorded significantly lower vibration values using the smartphone app and SEE.SENSE bicycle lights methods. In contrast, cobblestone-paved streets like Witte Nonnenstraat (CS2) and Raamstraat (CS1) showed higher vibration values. The consistency in results suggests that these tools (app and smart bicycle lights) are reliable for capturing the magnitude of vibrations on the cycling infrastructure.

The results were consistent across SEE.SENSE, DCI, and RMS, suggesting that these methods are reliable for comfort assessment based on the magnitude of vibrations on the cycling infrastructure. Previous research consistently reports that increased vibrations reduce cycling comfort [12,15,16]. The analysis of RMS and DCI values further supports these findings, with RMS values reflecting the intensity of vibrations with ISO 2631-1 standards experienced by cyclists and DCI providing an assessment for comfort. Streets, such as Witte Nonnenstraat (CS2) and Raamstraat (CS1), both cobblestone-paved streets, had higher RMS values (2.24 and 1.96, respectively). The RMS values were very low on asphalt and concrete-paved streets like Bonnefantenstraat (CS1). These results align with previous studies, which found that concrete pavements had lower RMS values, which provided greater comfort for cyclists [20]. Similarly, another research study found good correlations between RMS values and moderate severity levels of alligator, longitudinal, and transverse cracks, common pavement deterioration indicators [38]. These cracks result in rougher, uneven surfaces that increase vibrations, ultimately reducing ride comfort for cyclists. The cobblestone-paved streets in the study area have very uneven surfaces; hence, the RMS was very high.

Similarly, some studies have reported lower DCI values, indicating a rough and uncomfortable ride for cyclists [15,33]. For example, one study reported that the street segments that resemble cobblestones paved had DCI values between 0.3 and 0.6 [34]. In our study, the mean DCIs for a similar pavement type were lower. For example, Witte Nonnenstraat (CS2) and Raamstraat (CS1), both cobblestone-paved, are relatively low, at 0.39 and 0.41, respectively, while Maastrichterstraat (M2), which has a mixed pavement type but predominantly cobblestone surface, shows a mean DCI of 0.56. Another study reported a similar DCI for cobblestone-paved streets [15]. For example, the mean DCI values ranged from 0.31 to 0.38 for old small granite cobblestones.

Conversely, smoother streets like Kolonel Dusartplein (AS1), Bonnefantenstraat (C1), and Schrijnwerkersstraat (PT2) had high DCI values. The higher DCI values on smoother pavements are in line with the literature. Studies demonstrated that asphalt-paved streets have higher DCI values [15,33]. One study reported that the mean DCI for asphalt was 0.8132 [15]. In the same research, the mean value for worn asphalt was 0.7332, indicating that the DCI would vary depending on the surface’s roughness. In a study by Wage et al. [33], although the asphalt paved roads had the highest DCI (0.4192) among the studied streets, the values were still lower. The asphalted streets in the research were in bad condition.

The Wilcoxon Signed-Rank test results showed no statistically significant difference between the comfort assessments (each combination of three methods) obtained from the SEE.SENSE, DCI, and RMS methods. The lack of a significant difference, with *p*-values above 0.05, implies that while minor variations exist between the methods, their overall assessment of cycling comfort is consistent. This suggests that all three methods reliably reflect the comfort levels cyclists experience across different streets, with no one method offering a significantly distinct perspective. Additionally, the Friedman test verified these findings by indicating no statistically significant differences among the comfort categories provided by the three methods. With a *p*-value of 0.0724, the test results suggest that these methods yield comparable outcomes in evaluating cycling comfort, making them valuable tools for urban planners and policymakers in assessing and improving the cycling infrastructure.

Studies argue that the involvement of citizens in data collection represents a significant advancement in participatory urban planning [27,29]. Smartphone apps and smart bicycle lights offer the opportunity to involve citizens in data collection. As more bikeways are added to roads, applying maintenance treatments becomes essential to maintain safe and comfortable conditions. By allowing cyclists to contribute data on road conditions and maintenance needs, city authorities can gather more longitudinal and real-time data on the cycling infrastructure [42]. Research has shown that bicycle infrastructure monitoring can be achieved cost-effectively by providing less expensive accelerometer sensors to cyclists, such as smart bicycle lights [34]. This citizen-driven approach enhances data quality and scalability. Additionally, involving citizens encourages a sense of shared responsibility, as cyclists see their contributions as actively shaping the cycling environment, which can lead to greater public support and advocacy for sustainable urban mobility initiatives.

## 5. Conclusions

In recent years, there has been growing interest in assessing bicycle surface roughness due to its significant impact on bicyclist comfort and road maintenance. This study uses smartphone accelerometer sensors and smart bicycle lights to assess comfort on cycling paths. Furthermore, the consistency of assessments across the different methods under different pavement conditions was also examined. The study results highlight the relationship between road surface conditions and cycling comfort. This observation is consistent across all methods employed in the study, supporting the importance of maintaining smooth surfaces for enhancing cycling comfort and safety.

The results of the study also confirm the reliability of SEE.SENSE devices in assessing the comfort of cyclists. The results show no significant difference from previously established methods, such as DCI and RMS. Although each method (SEE.SENSE, DCI, and RMS) provides a distinct way of measuring cycling comfort, they all converge on similar conclusions about which streets offer less or more comfort to cyclists. The findings show that either tool can be used where necessary for a city to make better decisions regarding planning and managing cycling facilities. Advanced systems like IPBs are usually expensive and require more technical knowledge to combine multiple datasets from different sensors, while smartphone apps and the SEE.SENSE smart bicycle lights are reliable alternatives. These devices are advantageous compared to the commonly used system as they are cheaper, time-saving, and, more importantly, effective.

To improve cycling conditions, city authorities should consider smartphone apps and smart bicycle lights for data-gathering since these tools provide the scalability and ease of data collection. In addition, the data collected are useful for cycling comfort and monitoring. Smart bicycle lights offer the advantage of crowdsourced data, enabling continuous infrastructure monitoring over time so city authorities can better identify and prioritize maintenance needs for cycling paths. There are a few limitations connected to this research as this study does not consider the impact of individual bumps on cycling comfort, focusing instead on overall path vibrations. Bicyclists were instructed to maintain speed on the ridden path and avoid variations in cycling speed, which could influence comfort assessments, and is recommended to be considered in future research.

## Figures and Tables

**Table 1:** Streets selected for this study.

Street Name	Notation	Length	Pavement Type	Pavement Quality
Kolonel Dusartplein	AS1	230	Asphalt	Very good
Havermarkt	AS2	300	Asphalt	Good
Capucienenstraat	M1	70	Mixed (asphalt and cobblestone)	Good
Maastrichterstraat	M2	400	Mixed (cobblestone and paving tiles)	Bad
Minderbroedersstraat	M3	160	Mixed (asphalt and paving tiles)	Very Good
Bonnefantenstraat	C1	170	Concrete	Very Good
Melderstraat	C2	250	Concrete	Very Good
Sint-Jozefsstraat	C3	210	Concrete	Very Good
Persoonstraat	PT1	170	Paving Tiles	Good
Schrijnwerkersstraat	PT2	160	Paving Tiles	Very Good
Paardsdemerstraat	PT3	120	Paving Tiles	Very Good
Raamstraat	CS1	190	Cobblestone	Very Bad
Witte Nonnenstraat	CS2	230	Cobblestone	Very Bad
Badderijstraat	CS3	160	Cobblestone	Bad

**Table 2:** Smartphone Sensor Applications.

Name of Smartphone Application	Export Data to Other Formats	GPS Position	Multi Record (GPS + Acceleration)	Limitations	Cost of Use
Physics Toolbox Sensor Suite	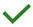	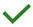	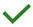	-	Free
Phyphox	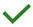	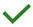	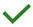	Does not export combined data	Free
Physics Toolbox Accelerometer	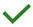	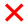	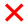	Does not export combined	Free
SensorLog	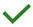	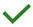	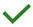	Needs to be purchased for use	5.99 Euro
Sensor Logger	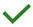	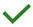	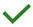	Needs pro version for exporting combined CSV	3.99 Euro per month
Sensors Toolbox-Multitool	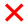	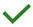	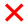	Does not export combined data	Free
EXA Sensors Toolbox	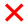	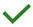	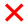	Needs a pro version for exporting data	1.99 Euro

**Table 3:** Comfort perception based on vibration.

Acceptable Values of Vibration Magnitudes for Comfort (m/s²) (ISO 2631-1)	Values Used [20]	Likely Users’ Reactions	Classification Used in This Study
Less than 0.315	Less than 0.315	Not uncomfortable	1
0.315–0.63	0.315–0.63	A little uncomfortable	2
0.5–1	0.63–1	Fairly uncomfortable	3
0.8–1.6	1–1.6	Uncomfortable	4
1.25–2.5	1.6–2.5	Very uncomfortable	
More than 2.5	More than 2.5	Extremely uncomfortable	5

## Data Availability

The raw data supporting the conclusions of this article will be made available by the authors upon request.
